# Description and Prediction of the Development of Metabolic Syndrome: A Longitudinal Analysis Using a Markov Model Approach

**DOI:** 10.1371/journal.pone.0067436

**Published:** 2013-06-20

**Authors:** Lee-Ching Hwang, Chyi-Huey Bai, San-Lin You, Chien-An Sun, Chien-Jen Chen

**Affiliations:** 1 Department of Family Medicine, Mackay Memorial Hospital, Taipei, Taiwan; 2 Mackay Medical College, New Taipei City, Taiwan; 3 School of Public Health, College of Public Health and Nutrition, Taipei, Taiwan; 4 Genomics Research Center, Academia Sinica, Taipei, Taiwan; 5 Department of Public Health, College of Medicine, Fu-Jen Catholic University, New Taipei, Taiwan; INRCA, Italy

## Abstract

**Background:**

Delineating the natural history of metabolic syndrome (MetS) is prerequisite to prevention. This study aimed to build Markov models to simulate each component’s progress and to test the effect of different initial states on the development of MetS.

**Methods:**

MetS was defined with revised AHA/NHLBI criteria. Each reversible multistate Markov chain consisted of 8 states (no component, five isolated component states, 2-component state, and MetS state). Yearly transition probabilities were calculated from a five-year population-based follow up studywhich enrolled 2,247 individuals with mean aged 32.4 years at study entry.

**Results:**

In men, high BP or a 2-component state was most likely to initiate the progress of MetS. In women, abdominal obesity or low HDL were the most likely initiators. Metabolic components were likely to occur together. The development of MetS was an increasing monotonic function of time. MetS was estimated to develop within 15 years in 12.7% of young men with no component, and 2 components developed in 16.3%. MetS was estimated to develop in 10.6% of women with at the age of 47, and 2 components developed in 14.3%. MetS was estimated to develop in 24.6% of men and 27.6% of women with abdominal obesity, a rate higher than in individuals initiating with no component.

**Conclusions:**

This modeling study allows estimation of the natural history of MetS. Men tended to develop this syndrome sooner than women did, i.e., before their fifth decade of life. Individuals with 1 or 2 components showed increased development of MetS.

## Introduction

Metabolic syndrome (MetS) is a precursor of cardiovascular disease and type 2 diabetes mellitus [1]. The burden of Mets and its component risk profile appears to be large and increases worldwide. Estimates suggest that MetS is present in at least a third of the population in most countries [2]. Several studies of incidence of MetS have reported. Those previous studies investigated the incidence of MetS and/or each component of MetS according to risk factors, such as older and less educated participants [3], smoking habit [4], alcohol consumption [5], high waist-to-hip ratio and fasting insulin levels [6]. Delineating the natural history of MetS is prerequisite to detecting high-risk groups and preventing its development. MetS is defined as the presence of three or more out of five components; therefore, there are 32 different states and1,024 transitions between states. It is difficult to establish which metabolic component leads to the cascade of disorders that characterize the syndrome. Haring et al. used the map generator to generate a network-based approach to model the progression of MetS components and their change [7]. However, many obstacles hinder the assessment of the natural history of MetS.The Markov model enables the collection, calculation, and summarization of data from groups with a wide variety of characteristics [8]. In the Markov model, the clinical states that individuals pass through are defined individually and are integrated in a system of transition probability from one state to another within a cycle. Markov models are well-recognized methods for simulating the natural history of chronic diseases [9]–[13]. Silverstein et al. applied a Markov model analysis of a population-based cohort to describe the clinical course of Crohn’s disease [13].

The Taiwanese Survey on the Prevalence of Hyperglycemia, Hyperlipidemia, and Hypertension (TwSHHH) was a valuable, large-sized, population-based study in Taiwan [14].The aim of this analysis of the application of Markov simulation was to describe the longitudinal course of the development of MetS and to determine gender differences in the natural history of MetS components and to test the effect of different initial states on the development of MetS.

## Materials and Methods

The basic data for this study came from the TwSHHH, a nation-wide cross-sectional survey that was conducted in 2002. A total of 6600 individuals had completed questionnaire interviews, blood pressure (BP) and other biomarker measurements. The follow-up program, named TwSHHH-II, was conducted in 2007 to estimate the incidences of cardiometabolic diseases for people in Taiwan. There were 4,682 individuals including in the TwSHHH-II, resulting in a response rate of 70.9%. The mean follow-up was 5.4 years. Details of the TwSHHH-II cohort were described previously [15]. The protocols for the TwSHHH were approved by the Institutional Review Board at the Bureau of Health Promotion, Department of Health, Executive Yuan, in Taiwan. All individuals gave written informed consent for participation.

Because the rates of cardiometabolic risk factors were significantly different in individuals aged over 50 years [14], data were limited from individuals aged 18–45 years for taking focus on young adult group. Therefore, individuals who were younger than 18 years (n = 293) or older than 45 years (n = 1,972), had a history of stroke or coronary heart disease (n = 79), or did not provide a blood specimen (n = 91) were excluded from this analysis, leaving data from 2247 participants (1,032 men and 1,215 women).

### Data Collection

Procedures and measurements were done during home visits. Demographic data were collected and the medical history was assessed. During the visit, sitting BP and anthropometric measurements were made. Waist circumference was measured with a inelastic tape measure placed parallel to the floor at the end of a relaxed expiration. Two BP measurements were taken using a random-zero mercury column sphygmomanometer in 2002 and an electric sphygmomanometer (BP3AC1-1, Microlife Cooperation, Berneck, Switzerland) in 2007. A third BP measurement was made if the first two BP readings differed by more than 10 mmHg. The average of the two closest readings was calculated and used in the analysis. Venous blood (20 ml) was taken for blood biochemistry and lipid profile. Biochemical variables, fasting total cholesterol (Lieberman-Burchard method), triglyceride (TG) (Bucolo method) and fasting plasma glucose (FPG) (glucose oxidase method) were measured by an automated system (Vitros 550/750, Johnson and Johnson, USA) in 2002. Total cholesterol (CV 1.03%), TG (CV 0.93%), and FPG (CV 1.32%) were measured with an automatic analyzer (TBA-200 FR, Toshiba Cooperation, Tokyo, Japan) in 2007. In addition, electrophoresis was performed to measure high density lipoprotein cholesterol (HDL, CV 2.52%) and low density lipoprotein cholesterol (CV 2.85%). Smoking status was categorized as current, past and never. Regular alcohol drinking was defined as consuming alcohol beverage at least once per week for at least 6 months.

### Metabolic Syndrome Criteria

There are five components in MetS. The criteria for the MetS were fulfilled if 3 or more of the following components were present [16]: (i) high BP: BP of more than 130/85 mmHg or use of antihypertensive agents; (ii) high FPG: FPG of more than 100 mg/dl or use of antidiabetic agents; (iii) high TG: fasting TG levels of more than 150 mg/dl; (iv) low HDL: HDL level of less than 40 mg/dl in men and less than 50 mg/dl in women; (v) abdominal obesity: waist circumference greater than 90 cm in Asian men or greater than 80 cm in Asian women.

### Markov Chain Models

**Figure 1:**
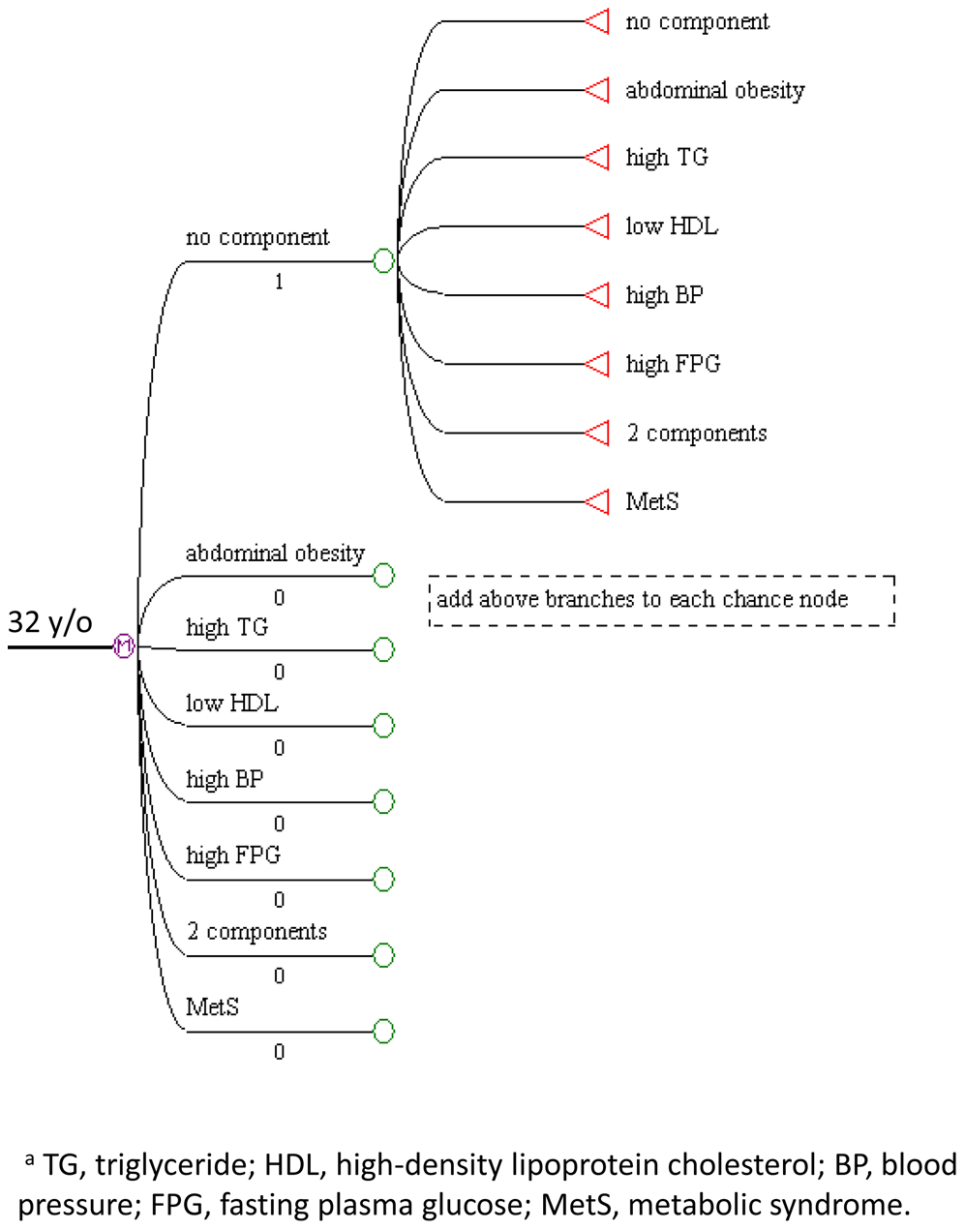
An 8-state Markov model to describe the progression of metabolic components.

Each Markov chain consisted of 8 states: no component state, isolated abdominal obesity state, isolated high TG state, isolated low HDL state, isolated high BP state, isolated high FPG state, 2-component state, and MetS state. The 2-component state indicated any two combinations of components. A graphical presentation of the Markov model is presented in Figure 1. These states are mutually exclusive and collectively exhaustive [8].

It was expected that individuals would experience a steady risk in the development of MetS over time. It was also expected that others would experience either steady improvement (resolution of MetS) or a highly variable course. Therefore, to capture all of these possible changes over time, we adopted a reversible multistate Markov model that allows for transitions to and from any state [9].

No component could transit into any “isolated component”, “2-component”, “MetS”, or maintain its steady state. Any isolated component could transit into “no component”, other “isolated component”, “2-component”, original “isolated component” or “MetS”. Two components state could transit into “no component”, “2-component”, any “isolated component” or “MetS”. MetS could reverse into other seven states or maintain its steady state.

During each Markov cycle, an individual in each gender group was assigned a probability of transitioning from one state to another. We analyzed our cohort survey data for annualized transitional probabilities. Evolution of MetS during the follow-up period was included in the simulation; therefore, there was no absorbing state in our Markov chains. At the start of the model simulation, the individual was assumed to have no component. Individuals were allowed to change their state every year, their outcome being determined by the probabilities defined at each chance node in the model. In addition, we constructed other Markov chains of different initial states, such as starting with isolated abdominal obesity, or starting with isolated high BP, and so on.

To simplify the model, a number of assumptions were necessary [8]. 1) The future progression depended only on a individual’s current state without any memory of prior states. 2) We assumed that each baseline transition rate remained constant throughout the follow-up period. 3) We also assumed that individuals with MetS could experience evolution in every cycle.

### Validation of the Model

To validate the MetS development of the model, we compared these outcomes with the empiric data. For the comparison between estimated prevalence of model and the empiric data, the time horizons of the model were set to 5 years to match the follow-up duration of the original cohort data.

### Statistical Analysis

SAS software (SAS Institute Inc, Cary, NC) was used for basic data analyses and calculation of annualized transition probabilities. Transition probabilities were calculated using a formula: P = 1−EXP^∧^(−rt); where P is the transition probability, r is the annual incidence rate and t is time. Differences in cross-tabulations were analyzed by using the Chi-square test. The Markov models were constructed using TreeAge pro 2008 software from Tree Age Software, Inc. (Williamstown, MA). The criterion for statistical significance was p<0.05.

## Results

Characteristics of the study individuals, stratified by gender are displayed in Table 1. Of 2,247 participants aged 18–45 years at study entry (in 2002), the mean age of the study cohort was 32.4 (SD: 8.0) years, and 54.1% were women. MetS developed in 164 participants who were without MetS at baseline examination, with incidence rates of 19.5/1000 person-years in men and 11.4/1000 person-years in women. MetS had reversed in 96 participants, with resolution rates of 79.8/1000 person-years in men and 72.0/1000 person-years in women during the 5.4-year follow-up.

**Table 1:** Basic characteristics of the study sample.

	Men		Women		
Basic surveya,b	N = 1032		N = 1215		*p*
Age (years)	32.1	±8.1	32.7	±7.9	0.0629
Body massindex (kg/m^2^)	24.0	±3.8	22.2	±3.6	<0.0001
Abdominalobesity	234	(22.7)	202	(16.6)	0.0003
High TG	317	(30.7)	144	(11.9)	<0.0001
Low HDL	222	(21.5)	314	(25.8)	0.0163
High BP	214	(20.7)	83	(6.8)	<0.0001
High FPG	97	(9.4)	92	(7.6)	0.1199
MetS in 2002	145	(14.1)	84	(6.9)	<0.0001
Currentsmoker (%)	541	(52.4)	77	(6.3)	<0.0001
Regular alcoholdrinker (%)	254	(24.6)	58	(4.8)	<0.0001

a Values for continuous characteristics are expressed as mean ± SD; values for categorical data were expressed as n (%).

b TG, triglyceride; HDL, high-density lipoprotein cholesterol; BP, blood pressure; FPG, fasting plasma glucose; MetS, metabolic syndrome.

### The Markov Chain Model and Transition Probabilities

The Markov chain model for the natural history of MetS depended on probabilities for transition between any 2 states at 1-year intervals. The results are shown as transition probabilities in Table 2 and Table 3. The probabilities for transition from the “no component” state, 2-component state, isolated high BP state, high FPG state, or high TG state to MetS were higher in men than in women. However, the probabilities for transition from the “no component” state to abdominal obesity or isolated low HDL were higher in women. MetS components always occurred simultaneously. In men, the progress of MetS components was likely to be initiated by high BP or the 2-component state, but in women, it was initiated by abdominal obesity or the low HDL state.

**Table 2:** Annualized transition probabilities (%) in Markov chain models for men.

		Event state							
Starting state	No component	abdominal obesity	high TG	low HDL	high BP	high FPG	2 components		MetS
No component	92.89	0.91	1.08	0.74	1.99	0.20	1.73		0.45
Isolated abdominal obesity	2.16	86.77	0.35	0.70	0.35	0.01	6.76		2.93
Isolated high TG^a^	4.65	0.25	84.35	0.25	0.75	0.25	6.89		2.62
Isolated low HDL	12.89	0.01	0.32	79.64	2.31	0.01	3.02		1.63
Isolated high BP	6.10	0.32	0.64	0.96	85.66	0.64	3.38		2.31
Isolated high FPG	11.29	0.90	1.84	0.00	1.84	79.41	3.84		0.90
2 components	2.27	1.29	1.17	0.69	0.81	0.58	86.33		6.87
MetS	0.52	0.65	0.26	0.13	0.91	0.26	6.14		91.14

a TG, triglyceride; HDL, high-density lipoprotein cholesterol; BP, blood pressure; FPG, fasting plasma glucose; MetS, metabolic syndrome.

**Table 3:** Annualized transition probabilities (%) in Markov chain models for women.

		Event state							
Starting state	No component	abdominal obesity	high TG	low HDL	high BP	high FPG	2 components		MetS
No component	92.82	1.67	0.32	2.73	0.41	0.16	1.49		0.41
Isolated abdominal obesity	2.22	85.42	0.00	0.27	0.01	0.01	6.39		5.70
Isolated high TG^a^	5.58	1.27	80.28	5.58	0.63	0.01	4.05		2.62
Isolated low HDL	10.24	0.66	0.11	85.23	0.54	0.01	3.11		0.54
Isolated high BP	4.23	0.01	0.79	2.44	83.91	0.01	6.19		2.44
Isolated high FPG	6.70	5.19	0.59	2.44	0.59	80.81	3.10		0.59
2 components	4.82	1.55	0.17	1.73	0.85	0.01	86.67		4.21
MetS	0.45	0.90	0.22	1.13	0.45	0.45	4.10		92.31

a TG, triglyceride; HDL, high-density lipoprotein cholesterol; BP, blood pressure; FPG, fasting plasma glucose; MetS, metabolic syndrome.

### Initiating with No Component

A representative component progression in this cohort of the “no component” state, divided by gender, is presented in Table 4. The development of MetS was an increasing monotonic function of time. After 15 years, MetS was estimated to develop in 12.7% (CI, 10.7–14.9) of young men with “no component” state which had started at the age of 32 years, and 2-component was estimated to develop in 16.3% (CI, 14.1–18.8). Only 46.7% of men remained steady in the “no component” state (Table 4). MetS was estimated to develop in 10.6% (CI, 8.8–12.7) of young women, and 2-component developed in 14.3% (CI, 12.2–16.7). Of note, 49.8% of women were steady in the no component state.

**Table 4:** The predictive development of metabolic syndrome and its components (%) in individuals starting with no component according to the Markov chain models.

		Young men				Young women			
		Year 1	Year 5	Year 10	Year 15	Year 1	Year 5	Year 10	Year 15
No component	100	92.89	71.88	56.15	46.71	92.82	72.30	57.89	49.81
Isolated abdominal obesity	0	0.91	3.30	4.68	5.28	1.67	5.77	7.78	8.41
Isolated high TG^a^	0	1.08	3.71	4.94	5.26	0.32	1.00	1.23	1.26
Isolated low HDL	0	0.74	2.40	3.02	3.09	2.73	9.13	11.85	12.41
Isolated high BP	0	1.99	6.80	8.91	9.32	0.41	1.50	2.19	2.52
Isolated high FPG	0	0.20	0.76	1.13	1.31	0.16	0.49	0.60	0.64
2 components	0	1.73	7.64	12.88	16.34	1.49	6.77	11.43	14.32
MetS	0	0.45	3.52	8.30	12.69	0.41	3.04	7.04	10.63

a TG, triglyceride; HDL, high-density lipoprotein cholesterol; BP, blood pressure; FPG, fasting plasma glucose; MetS, metabolic syndrome.

### Initiating with any Isolated Component or 2-component State

**Figure 2:**
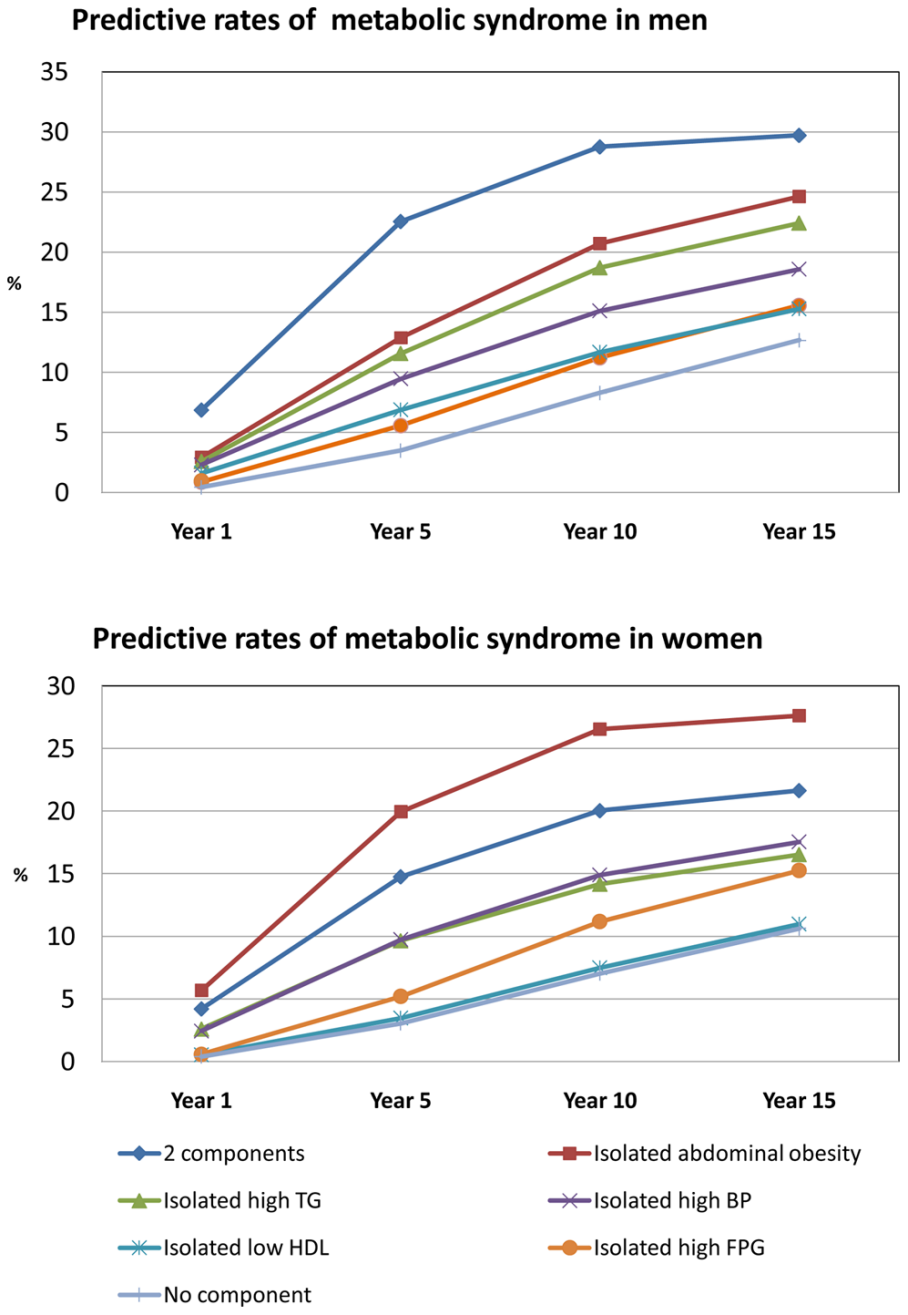
The predictive development of metabolic syndrome according to various starting component.

Individuals starting with any isolated component or 2-component state were more likely to develop MetS (Figure 2). MetS was estimated to develop after 15 years (at age 47 years) in 24.6% (CI, 22.0–27.4) of men and 27.6% (CI, 24.9–30.5) of women in whom the disease was initiated with the isolated abdominal obesity state. MetS was estimated to develop after 15 years in 29.7% (CI, 26.9–32.7) of men and 21.7% (CI, 19.1–24.3) of women in whom the disease was initiated with the 2-component state, a rate higher than in individuals in whom the disease initiated with the “no component” state. The results of this study suggest that MetS develops in more individuals who have 1 or 2 components.

Estimated prevalence of MetS after 5 years in men and women who had no component at study entry was 3.5% and 3.0%, respectively. We compared these outcomes with the empiric data for validation of model and found that estimated prevalence was similar with empiric data from original cohort in 2007 that showed 3.1% (CI, 1.7–5.1) in men and 2.5% (CI, 1.4–3.9) in women.

## Discussion

We developed a reversible multi-state Markov model and showed through simulation how these MetS components progress over 15 years in a young population. These results demonstrate that the main initiating components in men were high BP and a combination of 2 components. However, in women, the main isolated components were low HDL or abdominal obesity. Metabolic components were likely to occur together, which implies a close relation between these metabolic components.

Some researchers believe that high BP is not a main metabolic component. Macchia et al. showed that a clinically diagnostic score for MetS improves its predictivity of diabetes mellitus [17]. Their score system does not include a high BP component. Haring et al. generate a map of risk factor flow to visualize clustered MetS components and their change over a five-year follow-up period and identified hypertension and central obesity as the predominant risk factor cluster [7]. Our data revealed that in most young men MetS initiated with high BP. People with MetS are at increased risk of diabetes mellitus as well as at increased risk of cardiovascular events [18]. Hypertension is a well-defined risk factor for cardiovascular diseases. In our results, high BP components showed its important role in MetS, especially among young men.

The model initiated with any isolated MetS component or combination of 2 components accelerated the development of MetS in both genders; contrarily, the model also initiated with the “no component” state. Many researchers agree that obesity, dysglycemia, dyslipidemia, and hypertension coexist frequentlys [19], [20]. These MetS components have partially overlapping mechanisms of pathogenic actions mediated through common metabolic pathways. It should be specifically pointed out that individuals with 1 MetS component need to be examined for other components and have to be regularly followed up to identify the risk of development of MetS.

We found that women with abdominal obesity had an increased likelihood for incidences of a MetS event when compared with women with 2 components. Conceptually, MetS is a consequence of obesity and insulin resistance [21]. People with MetS usually pass through phases of excessive adipogenesis (obesity) [19]–[22]. MetS has been more prevalent in men than in women in recent years but has risen particularly in young women, where it is mainly driven by obesity [23].

In the absence of studies comprising metabolic components and longitudinal follow-up of large numbers of population, this Markov simulation modeling study allows estimation of the natural history of MetS. A Markov process is a frequently used mathematical model to describe disease progression over time [11], [24]–[27]. Sonnenberg and Naugler showed Markov models of influence in chronic liver disease [25]. Veenstra et al. constructed Markov processes that have been used to analyze hepatitis B virus infections [26]. Their results demonstrated that modeling the progression of a disease becomes more accurate than simple incidence rate presentation. Denton et al. constructed a Markov model with health states composed of cardiovascular events and metabolic factors to evaluate the optimal start time of statin treatment for different combinations of cardiovascular risk models and patient attributes [28]. Grassi et al. [29] analyzed data from a longitudinal study on a group of women with a history of gestational diabetes and constructed a three-state Markov chain model to represent the dynamics of changes between metabolic states. Our model with reversible chances (including resolution of MetS and its components) had the advantage of conceptualizing an intricate disease process.

This study had limitations. First, we estimated the natural history of MetS development, but it was not necessarily “natural” due to interventions of health services that were not taken into account in these analyses. Secondly, we assumed constant transition probability for following years, but probability might also increase with age [30]. On the contrary, increasing age was found to decrease the likelihood of resolution among individuals with MetS. Thirdly, we did not include medication use in high TG and low HDL components. The term “clean-blood agents” was represented as “lipid-lowering agents.” This concept is vague in Taiwan and would underestimate the high TG and low HDL components. Fourthly, mortality was not taken into account in this model. Slight overestimation of the cumulative incidence of MetS was anticipated. Decreased survival in persons with MetS is associated with the presence of other life-threatening diseases, such as diabetes mellitus and cardiovascular disease. Mortality rates for the general Taiwanese population were not included in these analyses and led to overestimation. This is especially true when gender differences are considered. Mortality rates in 2002 were 0.822% for middle-aged men and 0.385% for women. In this case, we assumed that the influence of mortality on the incidence of MetS is very small.

In conclusion, these results of this study suggest that men in whom MetS was initiated with no component tended to develop this syndrome sooner than women (i.e., before their fifth decade of life), but women with abdominal obesity develop this syndrome sooner than men. MetS develops in more individuals who have 1 or 2 components. The conclusions that can be drawn from our model are valid, in spite of some limitations, and the probability for bias is small. These findings confirm the importance of incorporating BP monitoring programs and weight control interventions into health services for assessing high-risk groups and preventing the development of MetS.
